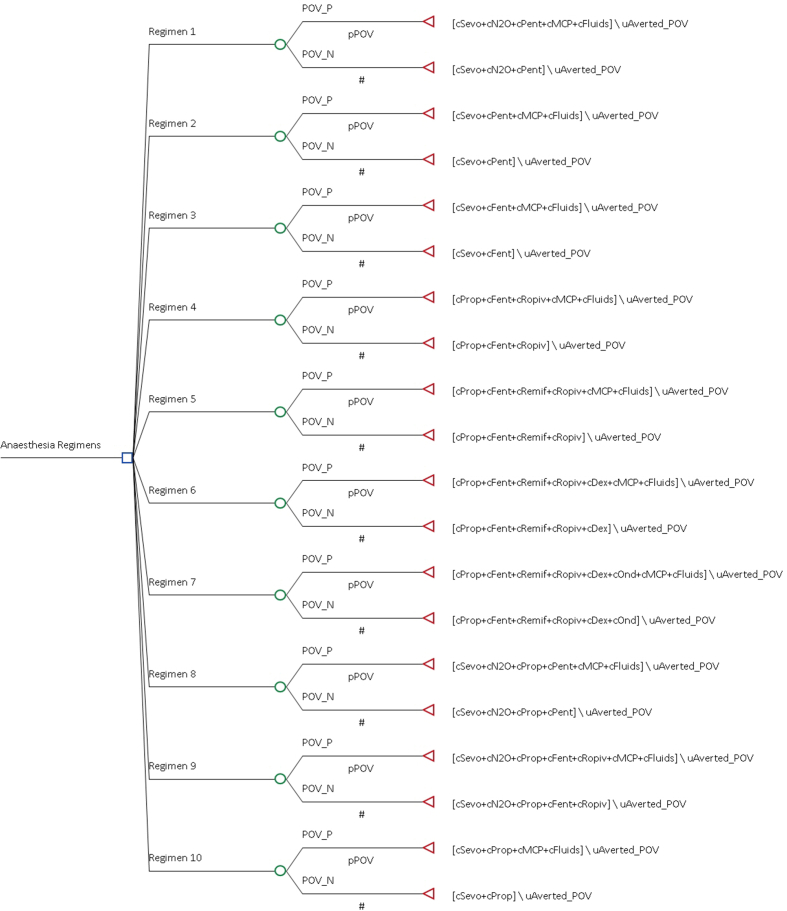# Cost-effectiveness analysis of anaesthesia regimens for paediatric strabismus surgery based on multicentre retrospective cohort data from Japan

**DOI:** 10.1016/j.bjao.2025.100404

**Published:** 2025-05-07

**Authors:** Soichiro Obara, Taiki Kojima, Yusuke Yamauchi, Takashi Fujiwara, Aya Sueda, Riku Takahashi

**Affiliations:** 1 Teikyo University Graduate School of Public Health, Tokyo, Japan; 2 Department of Anaesthesiology, Tokyo Metropolitan Ohtsuka Hospital, Tokyo, Japan; 3 Department of Anaesthesiology, Aichi Children's Health and Medical Centre, Obu, Japan; 4 Department of Comprehensive Paediatric Medicine, Nagoya University Graduate School of Medicine, Nagoya, Japan; 5 Department of Anaesthesiology, Hyogo Prefectural Kobe Children's Hospital, Kobe, Japan

**Keywords:** cost-effectiveness analysis, dexamethasone, general anaesthesia, ondansetron, paediatric strabismus surgery, postoperative nausea and vomiting, strabismus

## Abstract

**Background:**

Postoperative vomiting (POV) after paediatric strabismus surgery poses both clinical and economic challenges. This study evaluates the cost-effectiveness of different anaesthesia regimens, focusing on the addition of ondansetron (OND), in preventing POV.

**Methods:**

A cost-effectiveness analysis was conducted from the perspective of a Japanese public healthcare payer using retrospective cohort data of children (aged 3–15 years) undergoing strabismus surgery at three institutions (February 2016–November 2023). The primary outcome measure was cost per averted POV (aPOV) within 24 hours post-surgery. Incremental cost-effectiveness ratios were calculated, and sensitivity analyses accounted for uncertainties.

**Results:**

A total of 2772 patients were included. Total intravenous anaesthesia (TIVA) regimens were compared, as OND was used only in TIVA regimens. The aPOV rate for TIVA with sub-Tenon block and dexamethasone (DEX) was 91.0%, whereas TIVA with sub-Tenon block, DEX, and OND had an aPOV rate of 96.3%. The incremental cost of adding OND was €21.2, resulting in an incremental cost-effectiveness ratio of €400.6 per aPOV. A sensitivity analysis showed OND cost was the most influential factor. The cost-effectiveness acceptability frontier showed the probability of cost-effectiveness for TIVA+DEX ranged from 0% to 97.4% for thresholds of €0–€200 per aPOV, whereas TIVA+DEX+OND raged from 0% to 9.3%, and TIVA only ranged from 100% to 0%.

**Conclusions:**

TIVA with DEX alone is the most cost-effective regimen for preventing POV in paediatric strabismus surgery in the current Japanese public healthcare system. The addition of OND in TIVA regimens may not be justified from the viewpoint of cost-effectiveness.

## Introduction

Strabismus affects 2.15% of the Japanese population, with a higher incidence in school-aged children (5–9 yr: 6.02%) and older adults (75–79 yr: 2.61%).^1^ In the United States, 1.99% of children aged 0–5 yr and 1.37% of children aged 6–9 yr underwent strabismus surgery, with 1-yr re-operation rates of 5.13% and 3.95%, respectively.^2^ Given the high incidence, optimising anaesthesia is crucial, as strabismus surgery carries a significant risk of postoperative vomiting (POV), particularly in children aged 3 yr and older, for whom 5-hydroxytryptamine 3 receptor (5-HT_3_) antagonists are recommended.^3^, ^4^, ^5^

In 2021, Japan extended insurance coverage to 5-HT_3_ antagonists such as ondansetron (OND) for POV prevention in paediatric patients, building on their prior use for chemotherapy-induced nausea. Before this, traditional agents such as metoclopramide and dexamethasone (DEX) were used, but often lacked efficacy.^6^^,^^7^ Although 5-HT_3_ antagonists are effective in preventing POV, their higher cost raises questions about their cost-effectiveness compared with traditional treatments.^8^

Health Technology Assessment (HTA), formally introduced in Japan in 2019, helps to manage the rising costs of healthcare, ensure medical innovations are cost-effective, and maintain the sustainability of the universal health coverage in Japan.^9^^,^^10^ As Japan faces an aging population, the role of HTA in evaluating cost-effectiveness becomes increasingly important, particularly for higher-cost interventions.

Internationally, studies on the cost-effectiveness of OND for preventing POV in paediatric strabismus surgery have shown mixed results. Some suggest DEX alone offers better cost-effectiveness,^8^ whereas others favour OND.^11^ However, most previous studies used simplified models.^8^, ^11^, ^12^ Our study aims to address these gaps by conducting a comprehensive cost-effectiveness analysis of various anaesthesia regimens for paediatric strabismus surgery using real-world data and advanced methodologies, including deterministic and probabilistic sensitivity analyses. We specifically assess the cost-effectiveness of adding OND to traditional treatments as our primary objective, and compare the cost-effectiveness of different anaesthesia regimens as our secondary objective. By doing this, we hope to provide valuable evidence that could guide evidence-based healthcare resource allocation.

## Methods

### Study design

A decision-tree (DT) model was constructed to estimate and compare the health outcomes and costs associated with different anaesthesia regimens for paediatric strabismus surgery. The primary clinical outcome was POV within 24 h of surgery, excluding nausea. All surgeries were assumed to be inpatient procedures, in line with the standard care practices at the participating centres.

This study was approved by the Institutional Ethics Committee of Aichi Children's Health and Medical Centre as a multicentre collaborative retrospective cohort study (approval number 2022057; chair: Komei Ito), with approval from two additional participating institutions. Because of the retrospective and non-invasive nature of the study, informed consent was waived. A notice, including the option to opt out, was posted on the institution's website.

### Study population

The study included patients aged 3–15 yr who underwent strabismus surgery between February 2016 and November 2023 at the three institutions in Japan: Aichi Children's Health and Medical Centre, Hyogo Prefectural Kobe Children's Hospital, and Tokyo Metropolitan Otsuka Hospital. Exclusion criteria comprised refusal to participate, chromosomal abnormalities, desflurane administration, and American Society of Anaesthesiologists Physical Status Classification (ASA-PS) ≥3. Children younger than 3 yr were excluded because of differing anaesthesia reimbursement rates in Japan^13^ and distinct risk thresholds for POV in this age group.^4^^,^^5^

### Data acquisition

Data were retrospectively collected through chart reviews of electronic medical and anaesthesia records, including patient characteristics (age, sex, weight, ASA-PS), medical history (e.g. cerebral complications, chromosomal abnormalities), anaesthesia details (e.g. medication types and doses), and surgical factors (e.g. muscle manipulation, ocular blocks). All data were anonymised in compliance with relevant ethical guidelines.

### Anaesthesia regimens

Anaesthesia regimens were selected at the discretion of the anaesthetists at each institution. For the purposes of analysis, the regimens were categorised into five categories based on anaesthesia maintenance methods: Category 1, volatile maintenance anaesthesia (VMA) with nitrous oxide (N_2_O); Category 2, VMA without N_2_O; Category 3, total intravenous anaesthesia (TIVA); Category 4, combined intravenous and inhalation anaesthesia (CIVIA) with N_2_O; C5, CIVIA without N_2_O.

Additionally, the regimens were further classified based on the use of intraoperative opioids, antiemetics (e.g. metoclopramide, DEX, OND), and the performance of a sub-Tenon block by the ophthalmologist. The 10 most frequently used anaesthetic regimens across the participating centres were identified for analysis.

### Economic analysis

This study adhered to the Guideline for Economic Evaluation of Healthcare Technologies in Japan^14^ and the Consolidated Health Economic Evaluation Reporting Standards (CHEERS) 2022 Guidelines.^15^

### Time horizon

The time horizon for the cost-effectiveness analysis was limited to the first 24 h post-surgery, as this period encompasses the majority of POV episodes, which typically resolve within this timeframe. A 0% discount rate was applied to both costs and outcomes because of the short timeframe.

### Cost estimation

The analysis was conducted from a Japanese public healthcare payer perspective, which considered only direct healthcare costs, excluding indirect costs such as those related to family productivity losses because of POV. Direct costs included anaesthetic drugs, antiemetic medications, and treatments for POV. Drug costs were based on the Japanese Pharmaceutical Price List 2024.^16^ Surgery, anaesthesia, and medical staff fees were not separately calculated as they were assumed to be consistent across all anaesthesia regimens. For patients requiring rescue treatments for POV, costs for additional antiemetic medications (e.g. metoclopramide) and intravenous fluid therapy were incorporated into the cost calculations. It was assumed that all patients would be hospitalised overnight after surgery, in accordance with standard practice in the three participating hospitals, irrespective of whether they experienced POV. Intravenous cannulas were maintained *in situ* after surgery for all patients. The total costs were calculated using a micro-costing approach, where the unit costs for each medication and intervention were derived from data from the three institutions.

### Currency and price year

Currency conversion was calculated using the exchange rate of 1 JPY=€0.0065 as of December 7, 2024.

### Outcome (effectiveness)

The primary health outcome was the prevention of POV, as measured within the 24-h postoperative period. On the basis of data from the three hospitals and existing literature,^17^^,^^18^ the ‘averted POV’ (aPOV) was calculated using the formula aPOV=1−POV rate.

### Cost-effectiveness analysis

Cost-effectiveness was assessed by comparing the costs of different anaesthesia regimens with their effectiveness in preventing POV, using the incremental cost-effectiveness ratio (ICER).

Deterministic and probabilistic sensitivity analyses were conducted to account for data uncertainties. The primary sources of uncertainty in the model included POV incidence, medication costs, medication doses, and anaesthesia time. A Monte Carlo simulation was performed to account for uncertainty in the cost-effectiveness estimates, running 10 000 iterations to generate probability distributions for each anaesthesia regimen. The cost-effectiveness acceptability curve (CEAC) and frontier (CEAF) were used to assess the likelihood of cost-effectiveness across a range of willingness-to-pay (WTP) thresholds between €0 and €200 per aPOV.

The WTP threshold for preventing one episode of POV was set at €96.5 (interquartile range [IQR]: €44.9–€167.3) based on a previously published study,^19^ which reported that patients were willing to pay a median of US$56 (IQR: US$26–US$97) as of 2001. The current WTP value was adjusted for inflation using the annual changes in the US Consumer Price Index and converted from US$ to € using the relevant exchange rate as of December 7, 2024.

IBM SPSS Statistics v26 (IBM, Armonk, NY, USA) and TreeAge Pro Healthcare 2024 (TreeAge Software, LLC, Wilmington, MA, USA) were used for descriptive statistics, as well as cost-effectiveness modelling and analysis.

## Results

### Clinical data from chart review

Between February 2016 and November 2023, a total of 3571 children underwent strabismus surgery at the participating institutions. After applying the exclusion criteria (e.g. age <3 or >16 yr, second or subsequent strabismus surgeries, chromosomal abnormalities, ASA-PS ≥3, use of desflurane), 2772 patients were included in the analysis (Supplementary Fig. S1). Detailed anaesthesia regimens and POV incidences for each regimen are provided in Supplementary Table S1.

### Top 10 anaesthesia regimens

The 10 most frequently used anaesthesia regimens are summarised in Table 1, with corresponding patient numbers and POV incidences. In total, 2222 patients (80.2% of eligible patients) received one of these regimens. Notably, OND was only administered in a TIVA regimen with use of propofol, fentanyl, and remifentanil. Descriptive statistics for age, weight, anaesthesia and surgery durations, and medication dosages are presented in Supplementary Table S2. A DT model was developed to compare the cost-effectiveness of these regimens (Supplementary Fig. S2).

**Table 1:** Top 10 anaesthesia regimens included in the analysis. CI, confidence interval; CIVIA, combined inhalational and intravenous anaesthesia; DEX, dexamethasone; i.v., intravenous; N_2_O, nitrous oxide; OND, ondansetron; POV, postoperative vomiting; TIVA, total intravenous anaesthesia; VMA, volatile maintenance anaesthesia.

Eligible anaesthesia regimens for the cost-effective analysis in this study	Anaesthetics for maintenance	Intraoperative use of opioids (i.v.)	Prophylactic use of antiemetic medications	Application of sub-Tenon block by ophthalmologists (yes/no)	Number of patients	Number of patients suffering POV within 24 h	Incidence of POV (95% CI)	
Regimen 1	Category 1: VMA with N_2_O	Pentazocine	None	No	157	49	0.312	(0.261–0.363)
Regimen 2	Category 2: VMA without N_2_O	Pentazocine	None	No	146	34	0.233	(0.181–0.285)
Regimen 3		Fentanyl	None	No	59	7	0.119	(0.057–0.181)
Regimen 4	Category 3: TIVA	Fentanyl	DEX	Yes	61	4	0.066	(0.016–0.116)
Regimen 5		Fentanyl and remifentanil	None	Yes	75	14	0.187	(0.126–0.248)
Regimen 6		Fentanyl and remifentanil	DEX	Yes	1229	111	0.09	(0.079–0.101)
Regimen 7		Fentanyl and remifentanil	DEX, OND	Yes	326	12	0.037	(0.016–0.058)
Regimen 8	Category 4: CIVIA with N_2_O	Pentazocine	None	No	44	11	0.25	(0.131–0.369)
Regimen 9		Fentanyl	DEX	Yes	88	6	0.068	(0.014–0.122)
Regimen 10	Category 5: CIVIA without N_2_O	No opioids	None	No	37	4	0.108	(0.031–0.185)

Note: Definition of anaesthesia regimens 1–10 used in all relevant tables and figures. Regimen 1: VMA with N₂O, pentazocine, no prophylactic antiemetics, no sub-Tenon block. Regimen 2: VMA without N₂O, pentazocine, no prophylactic antiemetics, no sub-Tenon block. Regimen 3: VMA without N₂O, fentanyl, no prophylactic antiemetics, no sub-Tenon block. Regimen 4: TIVA, fentanyl, dexamethasone, with sub-Tenon block. Regimen 5: TIVA, fentanyl & remifentanil, no prophylactic antiemetics, with sub-Tenon block. Regimen 6: TIVA, fentanyl & remifentanil, dexamethasone, with sub-Tenon block. Regimen 7: TIVA, fentanyl & remifentanil, dexamethasone and ondansetron, with sub-Tenon block. Regimen 8: CIVIA with N₂O, pentazocine, no prophylactic antiemetics, no sub-Tenon block. Regimen 9: CIVIA with N₂O, fentanyl, dexamethasone, with sub-Tenon block. Regimen 10: CIVIA without N₂O, no opioids, no prophylactic antiemetics, no sub-Tenon block.

### Anaesthesia regimens in primary analysis

The primary analysis focused on the TIVA regimen with use of propofol, fentanyl, and remifentanil, because OND was exclusively used in this specific regimen in our clinical data. Therefore, three regimens were included in the primary analysis: Regimen 5 (TIVA with remifentanil, fentanyl, and no antiemetics), Regimen 6 (TIVA with fentanyl, remifentanil, and DEX), and Regimen 7 (TIVA with fentanyl, remifentanil, DEX, and OND). Seventy-five patients received Regimen 5, 1229 received Regimen 6, and 326 received Regimen 7. All regimens included a sub-Tenon block. The lowest POV incidence was observed with Regimen 7 at 3.7% (95% confidence interval [CI]: 1.6–5.8%).

### Anaesthesia regimens in secondary analysis

The secondary analysis incorporated all 10 anaesthesia regimens, including VMA, TIVA, and CIVIA techniques (Table 1). Regimen 1 (VMA with N_2_O and pentazocine) had the highest POV incidence at 31.2% (95% CI: 26.1–36.3%), whereas Regimen 7 (TIVA+DEX+OND) had the lowest at 3.7% (95% CI: 1.6–5.8%).

### Cost and utility analyses

Cost and utility data derived from the chart review are summarised in Table 2 and Supplementary Table S2.

**Table 2:** Key input parameters, ranges in one-way sensitivity analyses, and distributions used in probabilistic sensitivity analyses. The input parameters related to anaesthesia time and the dose of each drug per anaesthesia regimen (1–10) for the model are presented in Supplementary Table S2. For these parameters, the ranges used in the one-way sensitivity analyses correspond to the interquartile range (25th to 75th percentile), and the distributions used in the probabilistic sensitivity analyses were based on a gamma distribution. CI, confidence interval; inh., inhalational; i.v., intravenous; POV, postoperative vomiting; WTP, willingness to pay.

Parameter	Unit	Value	Range	Distribution
POV probabilities				
Regimen 1		0.312	95% CI: 0.239–0.384	Beta
Regimen 2		0.233	95% CI: 0.189–0.477	Beta
Regimen 3		0.119	95% CI: 0.052–0.327	Beta
Regimen 4		0.066	95% CI: 0.003–0.226	Beta
Regimen 5		0.187	95% CI: 0.141–0.371	Beta
Regimen 6		0.090	95% CI: 0.080–0.111	Beta
Regimen 7		0.037	95% CI: 0.025–0.075	Beta
Regimen 8		0.250	95% CI: 0.169–0.477	Beta
Regimen 9		0.068	95% CI: 0.018–0.173	Beta
Regimen 10		0.108	95% CI: 0.001–0.243	Beta
Drug costs∗^,^†		(€)	(€)	
Sevoflurane (inh.)‡	ml	0.177	-	Gamma
Nitrous oxide (inh.)¶	g	0.023	0.016–0.023	Gamma
Propofol (i.v.)	mg	0.0244	0.0193–0.0244	Gamma
Pentazocine (i.v.)	15 mg	0.579	-	Gamma
Fentanyl (i.v.)	μg	0.01645	0.01573–0.01645	Gamma
Remifentanil (i.v.)	μg	0.005717	0.003039–0.005717	Gamma
Metoclopramide (i.v.)	10 mg	0.377	0.371–0.377	Gamma
Dexamethasone (i.v.)	6.6 mg	1.404	1.281–1.404	Gamma
Ondansetron (i.v.)	4 mg	21.379	8.775–21.379	Gamma
Ropivacaine	10 ml	3.380	1.866–3.380	Gamma
Fluids (i.v.)	500 ml	1.378	1.378–2.366	Gamma
Procedure costs				
General anaesthesia§		390	–	Gamma
Sub-Tenon block||		–	–	–
Utilities				
Averted POV		1−POV probability	–	–
WTP threshold#		96.5		–

Note: Definition of anaesthesia regimens 1–10 used in all relevant tables and figures. Regimen 1: VMA with N₂O, pentazocine, no prophylactic antiemetics, no sub-Tenon block. Regimen 2: VMA without N₂O, pentazocine, no prophylactic antiemetics, no sub-Tenon block. Regimen 3: VMA without N₂O, fentanyl, no prophylactic antiemetics, no sub-Tenon block. Regimen 4: TIVA, fentanyl, dexamethasone, with sub-Tenon block. Regimen 5: TIVA, fentanyl & remifentanil, no prophylactic antiemetics, with sub-Tenon block. Regimen 6: TIVA, fentanyl & remifentanil, dexamethasone, with sub-Tenon block. Regimen 7: TIVA, fentanyl & remifentanil, dexamethasone and ondansetron, with sub-Tenon block. Regimen 8: CIVIA with N₂O, pentazocine, no prophylactic antiemetics, no sub-Tenon block. Regimen 9: CIVIA with N₂O, fentanyl, dexamethasone, with sub-Tenon block. Regimen 10: CIVIA without N₂O, no opioids, no prophylactic antiemetics, no sub-Tenon block.

∗ Costs were converted to 2024 Euro from Japanese Yen as of December 7, 2024 (1 JPY=€0.0065).

† The drug costs were calculated according to the National Health Insurance Drug Price Standard, Drug Price Standard Score Chart, April 2024 version, Tokyo, Japan, Social Insurance Research Laboratory, 2024. In the scenario analyses, drug costs were calculated based on the originator drug, whereas in the one-way sensitivity analysis, the price difference between the originator and generic drugs was used as the range for drug costs. However, if there was no price difference between the originator and generic drugs, no range was set.

‡ Consumption rate (millilitres per hour)=3.3 (sevoflurane coefficient)×concentration (%)×gas flow rate (litres per minute). For example, on an hourly basis, the calculation is as follows: 3.3×2.5 (%)×3 (L min^−1^)=24.75 (ml). Therefore, the cost is calculated as 24.75 (ml)×0.177 (€ ml^−1^)=4.381 (€).

¶ The flow rate of nitrous oxide (litres per minute) is calculated using the following formula (approximate value): nitrous oxide flow rate (litres per minute)=nitrous oxide mass (grams)×0.51 (L)÷anaesthesia duration (minutes) (note: 1 g of nitrous oxide corresponds to a volume of 0.51 L). Therefore, for example, at a flow rate of 1.5 (L min^−1^) for 60 min, using a concentration of 50% and a total flow rate of 3 (L min^−1^), the cost is calculated as follows: 1.5 (L min^−1^)×60 (min)÷0.51×0.023 (€ g^−1^)=4.059 (€ h^−1^).

§ As per the Japanese national medical fee schedule as of December 7, 2024, if the duration of general anaesthesia exceeds 2 h, an additional charge for anaesthesia management is applied. For every additional 30 min or a portion thereof, €39 (6000 JPY) is added to the specified fee.

|| In Japan, the procedural fee cannot be billed for local infiltration anaesthesia performed by a surgeon under general anaesthesia, whereas the cost of medications used can be billed.

# Because of the absence of Japanese-specific WTP values for POV prevention (averted POV), the estimate from previously published study was extrapolated and adjusted for both currency and price year.^19^

### Scenario analysis for primary objective

The base case costs, utilities, and cost-effectiveness measures for evaluating the addition of antiemetic prophylaxis in TIVA are presented in Table 3. The aPOV rate was 91.0% for Regimen 6 (TIVA with DEX alone) and 96.3% for Regimen 7 (TIVA with DEX+OND), compared with 81.3% for Regimen 5 (TIVA without antiemetics). The incremental cost of adding OND to DEX was €21.23, resulting in an ICER of €400.60 per aPOV. Regimen 6 (TIVA including sub-Tenon block with DEX) was the most cost-effective at the WTP threshold, although Regimen 7 (TIVA+DEX+OND) was the most effective in preventing POV.

**Table 3:** Scenario analysis for primary objective. NMB represents the monetary value of the effectiveness of each anaesthesia regimen relative to its cost, calculated using a WTP threshold of €96.5 per aPOV. NMB was calculated as NMB=(Effectiveness×WTP)−Cost. Positive NMB values indicate a favourable balance of cost and effectiveness, with higher values reflecting more cost-effective strategies. aPOV, averted postoperative vomiting; DEX, dexamethasone; ICER, incremental cost-effectiveness ratio; NMB, net monetary benefit; TIVA, total intravenous anaesthesia; OND, ondansetron; WTP, willingness to pay.

Strategy	Cost (€)	Incremental cost (€)	Effectiveness (aPOV)	Incremental effectiveness (aPOV)	ICER	NMB
Regimen 5: TIVA	11.28	–	0.813	–	–	67.19
Regimen 6: TIVA+DEX	12.42	1.14	0.910	0.097	11.75	75.51
Regimen 7: TIVA+DEX+OND	33.65	21.23	0.963	0.053	400.57	59.38

### Sensitivity analysis for primary objective

A one-way sensitivity analysis comparing the ICER of Regimen 6 (TIVA+DEX) *vs* Regimen 7 (TIVA+DEX+OND) revealed that the ICER was most sensitive to the POV rate in Regimen 7 (TIVA+DEX+OND), followed by the cost of OND and the POV rate in Regimen 6 (TIVA+DEX) (Supplementary Fig. S3). The ICER scatterplot demonstrated that Regimen 6 (TIVA+DEX) was more cost-effective in the majority of simulations, with 90.12% of simulations falling in Quadrant III, where the intervention is less effective and less costly (Supplementary Fig. S4). A small proportion (9.87%) of simulations fell in Quadrant IV, where Regimen 6 is superior (more effective, less costly). These results suggest that Regimen 6 (TIVA+DEX) was more cost-effective in most scenarios compared with Regimen 7 (TIVA+DEX+OND).

**Fig 1:**
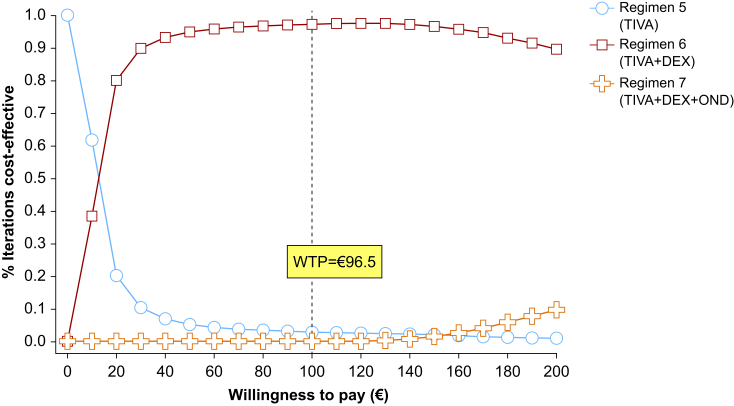
Cost-effectiveness acceptability curves (CEFCs) for probabilistic sensitivity analysis comparing TIVA regimens. The CEFC showed that TIVA+DEX was the most cost-effective in 97.0% of cases at a WTP threshold of €96.5 per averted postoperative vomiting, followed by TIVA alone (3.0%) and TIVA+DEX+OND (0%). DEX, dexamethasone; OND, ondansetron; TIVA, total intravenous anaesthesia; WTP, willingness to pay.

The probability of cost-effectiveness for TIVA+DEX varied from 0% to 97.4% for thresholds between €0 and €200 per aPOV, whereas TIVA alone ranged from 100% to 0.8% and TIVA+DEX+OND varied from 0% to 9.3% (Fig. 1). The CEAF revealed that TIVA+DEX was the most cost-effective in 97.0% of cases at a WTP threshold of €96.5 per aPOV, followed by TIVA alone (3.0%) and TIVA+DEX+OND (0%) (Fig. 1).

### Scenario analysis for secondary objective

**Fig 2:**
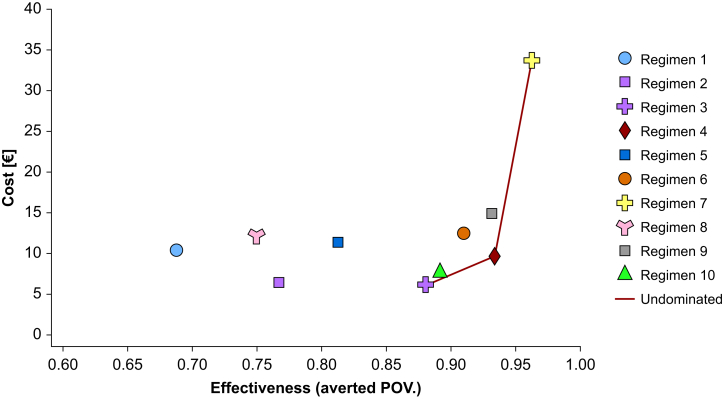
Efficiency frontier comparing all the top 10 regimens. The line which connects Regimen 3 (volatile maintenance anaesthesia without nitrous oxide, with fentanyl, but without prophylactic antiemetics or sub-Tenon block), Regimen 4 (TIVA with propofol, fentanyl, DEX, and sub-Tenon block), and Regimen 7 (TIVA+DEX+OND) represents the cost-effectiveness boundary, known as the efficiency frontier. The area on the left side of the efficiency frontier represents reduced cost-effectiveness. ‘Domination’ refers to situations where one regimen is both less costly and more effective than another in cost-effectiveness analysis. Regimens 3, 4, and 7 dominate the other seven regimens, meaning these three offer better value for money in terms of averted postoperative vomiting at a lower cost compared with the other treatments. DEX, dexamethasone; OND, ondansetron; POV, postoperative vomiting; TIVA, total intravenous anaesthesia. Note: Definition of anaesthesia regimens 1–10 used in all relevant tables and figures. Regimen 1: VMA with N₂O, pentazocine, no prophylactic antiemetics, no sub-Tenon block. Regimen 2: VMA without N₂O, pentazocine, no prophylactic antiemetics, no sub-Tenon block. Regimen 3: VMA without N₂O, fentanyl, no prophylactic antiemetics, no sub-Tenon block. Regimen 4: TIVA, fentanyl, dexamethasone, with sub-Tenon block. Regimen 5: TIVA, fentanyl & remifentanil, no prophylactic antiemetics, with sub-Tenon block. Regimen 6: TIVA, fentanyl & remifentanil, dexamethasone, with sub-Tenon block. Regimen 7: TIVA, fentanyl & remifentanil, dexamethasone and ondansetron, with sub-Tenon block. Regimen 8: CIVIA with N₂O, pentazocine, no prophylactic antiemetics, no sub-Tenon block. Regimen 9: CIVIA with N₂O, fentanyl, dexamethasone, with sub-Tenon block. Regimen 10: CIVIA without N₂O, no opioids, no prophylactic antiemetics, no sub-Tenon block.

The cost-effectiveness plane revealed an efficiency frontier connecting Regimen 3 (VMA without N_2_O, with fentanyl, but without prophylactic antiemetics or sub-Tenon block), Regimen 4 (TIVA with propofol, fentanyl, DEX, and sub-Tenon block), and Regimen 7 (TIVA+DEX+OND) (Fig. 2). These regimens dominated the other seven regimens, demonstrating a better balance of effectiveness and cost (Table 4, Fig. 2). Among the top 10 regimens, Regimen 4 (TIVA with propofol, fentanyl, DEX, and sub-Tenon block) was the most cost-effective with the highest net-monetary benefit (Table 4, Fig. 2).

**Table 4:** Scenario analysis for secondary objective. Regimens 3, 4, and 7 dominate the other seven regimens, meaning these three offer better value for money in terms of aPOV at a lower cost compared with the other treatments. Regimen 4 was selected as the benchmark because it represented the most cost-effective among the top 10 regimens. NMB represents the monetary value of the effectiveness of each anaesthesia regimen relative to its cost, calculated using a WTP threshold of €96.5 per aPOV. NMB was calculated as NMB=(Effectiveness×WTP)−Cost. Positive NMB values indicate a favourable balance of cost and effectiveness, with higher values reflecting more cost-effective strategies. aPOV, averted postoperative vomiting; Abs., absolutely; Ext., extendedly; DEX, dexamethasone; ICER, incremental cost-effectiveness ratio; NMB, net monetary benefit; TIVA, total intravenous anaesthesia; OND, ondansetron; WTP, willingness to pay.

Strategy	Cost (€)	Incremental Cost (€) (*vs* Regimen 4)	Effectiveness (aPOV)	Incremental Effectiveness (aPOV) (*vs* Regimen 4)	ICER	NMB	Dominance
Regimen 1	10.36	0.80	0.688	−0.246	−3.25	52.59	Abs. dominated
Regimen 2	6.38	−3.18	0.767	−0.167	19.04	63.80	Abs. dominated
Regimen 3	6.07	−3.49	0.881	−0.053	65.85	74.54	Undominated
Regimen 4	9.56	-	0.934	-	-	75.90	Undominated
Regimen 5	11.28	1.72	0.813	−0.121	−14.21	63.11	Abs. dominated
Regimen 6	12.42	2.86	0.91	−0.024	−119.17	70.85	Abs. dominated
Regimen 7	33.65	24.10	0.963	0.029	831.03	54.46	Undominated
Regimen 8	12.09	2.53	0.75	−0.184	−13.75	56.54	Abs. dominated
Regimen 9	14.88	5.32	0.932	−0.002	−2660.00	70.40	Abs. dominated
Regimen 10	7.56	−2.00	0.892	−0.042	47.62	74.06	Ext. dominated

Note: Definition of anaesthesia regimens 1–10 used in all relevant tables and figures. Regimen 1: VMA with N₂O, pentazocine, no prophylactic antiemetics, no sub-Tenon block. Regimen 2: VMA without N₂O, pentazocine, no prophylactic antiemetics, no sub-Tenon block. Regimen 3: VMA without N₂O, fentanyl, no prophylactic antiemetics, no sub-Tenon block. Regimen 4: TIVA, fentanyl, dexamethasone, with sub-Tenon block. Regimen 5: TIVA, fentanyl & remifentanil, no prophylactic antiemetics, with sub-Tenon block. Regimen 6: TIVA, fentanyl & remifentanil, dexamethasone, with sub-Tenon block. Regimen 7: TIVA, fentanyl & remifentanil, dexamethasone and ondansetron, with sub-Tenon block. Regimen 8: CIVIA with N₂O, pentazocine, no prophylactic antiemetics, no sub-Tenon block. Regimen 9: CIVIA with N₂O, fentanyl, dexamethasone, with sub-Tenon block. Regimen 10: CIVIA without N₂O, no opioids, no prophylactic antiemetics, no sub-Tenon block.

### Probabilistic sensitivity analysis for secondary objective

The CEAF for secondary analysis showed that the probability of cost-effectiveness for Regimen 4 (TIVA with propofol, fentanyl, DEX, and sub-Tenon block) varied from 0% to 58.4% for thresholds between €0 and €200 per aPOV, whereas Regimen 3 (VMA without N_2_O, with fentanyl, but without prophylactic antiemetics or sub-Tenon block) ranged from 98.6% to 9.4%, and Regimen 10 (CIVIA without N_2_O, opioids, antiemetics, or sub-Tenon block) ranged from 0% to 27.1%. Regimen 4 was the most cost-effective in 51.5% of simulations, followed by Regimen 3 (25.0%) and Regimen 10 (19.6%) at the WTP threshold of €96.5 per aPOV (Supplementary Fig. S5).

## Discussion

Our study provides insights into the cost-effectiveness of prophylactic antiemetic medications, specifically DEX and OND, in combination with TIVA and sub-Tenon block in paediatric strabismus surgery. Our findings suggest that DEX is the most cost-effective option for preventing POV in this context. The addition of OND, although beneficial in reducing POV, resulted in a higher ICER because of its higher cost. This is consistent with previous studies that demonstrated the cost-effectiveness of DEX in reducing POV, where DEX alone was found to be as effective as OND, but at a lower cost.^8^

Our clinical data showed relatively low POV rates with TIVA combined with sub-Tenon block in paediatric strabismus surgery. The POV rate was 18.7% (95% CI: 12.6–24.8%) with TIVA plus sub-Tenon block, 9.0% (95% CI: 7.9–10.1%) with TIVA plus sub-Tenon block and DEX (150 μg kg^−1^), and further reduced to 3.7% (95% CI: 1.6–5.8%) when OND was added. Historically, TIVA plus sub-Tenon block, DEX, and OND has led to substantially lower POV rates. Previous studies have shown that combining OND with DEX significantly reduces POV in paediatric strabismus surgery. For instance, low-dose OND (50 μg kg^−1^) with DEX (150 μg kg^−1^) reduced POV to 9%, compared with 28% with high-dose OND alone,^20^ and OND+DEX resulted in a POV rate of 10%, compared with 33.3% with OND alone.^21^ Another study observed a reduction in POV to 5% with OND+DEX, compared with 23% with DEX alone.^22^ Our findings showed minimal difference in POV rates between the TIVA+DEX and TIVA+DEX+OND regimens. The addition of sub-Tenon block may contribute to this further reduction. Recent meta-analyses suggest that sub-Tenon block helps reduce both POV and early postoperative pain by modulating ocular and visceral pain pathways.^23^ Although we did not conduct a specific cost-effectiveness analysis of sub-Tenon block, our clinical data suggest that combining TIVA with sub-Tenon block could be effective in reducing POV, warranting further investigation into its cost-effectiveness.

Our secondary analysis, which compared 10 anaesthesia regimens, found that the most cost-effective approach was TIVA with propofol, fentanyl, DEX, and sub-Tenon block (Regimen 4). This finding aligns with existing evidence supporting the use of multimodal anaesthesia, combining TIVA, DEX, and peri-bulbar anaesthesia for enhanced efficacy and cost-effectiveness.^23^^,^^24^ This approach demonstrates clear economic advantages in reducing POV compared with simpler regimens. Despite the apparent benefits of TIVA in reducing POV, it is important to note that the higher cost of TIVA regimens may limit their wider adoption. A previous study found that TIVA was more than three times as costly as inhalation anaesthesia, with only minor reductions in post-anaesthesia care unit (PACU) stay and no significant difference in hospital stay duration.^25^ Similarly, a systematic review suggested that single pharmacological prophylaxis could be equally effective as TIVA in preventing POV in paediatric patients.^26^ These findings imply that simpler and more cost-effective options could be considered in appropriate clinical settings. This raises the question of whether the additional cost of TIVA is justifiable when effective alternatives are available.

In our study, aPOV was used as the primary measure of effectiveness for antiemetic interventions. Several studies have supported using POV rates in paediatric settings as a valid measure for evaluating the cost-effectiveness of antiemetic drugs.^17^^,^^18^ Also, the brief and transient nature of POV is unlikely to have a long-term effect on overall quality of life (QOL). However, POV rates alone do not capture the broader impact of postoperative complications on the QOL. Measuring QOL in paediatric patients presents challenges as a result of developmental impacts on self-reporting accuracy and the need for age-specific measures. Therefore, future studies could consider incorporating broader measures, such as disability-adjusted life years (DALYs), to quantify the overall burden of postoperative complications. Such measures have been demonstrated as feasible in paediatric studies for other types of surgery, such as inguinal hernia and intestinal atresia repairs, although they have not yet been used for POV.^27^

Our study has several strengths. The multicentre design, incorporating data from three institutions, enhances the external validity and relevance of the findings for similar clinical settings. The use of individual patient-level data allowed for a detailed analysis of various anaesthesia regimens, providing insights applicable to clinical practice. Although the results may not be directly applicable to healthcare systems outside Japan, the methodological approach offers a valuable model for anaesthetists and policymakers seeking to optimise cost-effectiveness in paediatric anaesthesia.^28^ Future research should adapt this methodology to different healthcare systems, considering local cost structures and priorities.

Despite its strengths, our study has several limitations. First, the retrospective design and anaesthetist discretion in selecting anaesthesia regimens may have introduced bias, limiting generalisability. Although patient-level data allowed for detailed analysis, variation in anaesthetist choices could affect broader applicability. Additionally, the inclusion of OND only in TIVA regimens limited our ability to evaluate other anaesthesia combinations. Second, the cost of OND is likely to decrease over time, making its use more cost-effective. If the cost decreases in the future, this may influence the cost-effectiveness outcomes. To address this, we conducted a sensitivity analysis using a gamma distribution of the cost, capturing a broader spectrum of cost variations and strengthening the robustness of our findings. Third, we excluded surgical factors such as manipulated muscles, which affect POV rates.^29^^,^^30^ Anaesthetists at the participating institutions did not adjust anaesthesia based on the factor, limiting the accuracy of our cost-effectiveness analysis. Fourth, although we assumed all patients would be hospitalised overnight after surgery, we did not collect data on length of hospital stay or PACU stay, both of which can influence healthcare costs and patient outcomes. These factors were not the focus of this study but could provide valuable insights in future research. Fifth, we did not include certain cost factors, such as linen and nursing fees related to POV management, which may vary across institutions. These costs could introduce uncertainty into the analysis so that our study did not consider. Sixth, although we accounted for inflation, the WTP threshold used in our analysis was based on a US study sampling an adult population.^19^ The transferability of this WTP threshold to the Japanese healthcare context is uncertain, as WTP thresholds can vary across countries because of different healthcare priorities and economic conditions. To address this, we used the CEAC, which evaluates cost-effectiveness across a range of WTP values, allowing for greater flexibility in the analysis. However, future research could consider Japan-specific WTP thresholds or directly assess WTP in the Japanese population to further refine the cost-effectiveness analysis. Finally, we relied on the existing clinical data and the sensitivity analyses to ensure the robustness of our estimates but did not conduct a formal sample size calculation.Although a sample size calculation has gained recognition in recent years,^31^ it was not routinely performed in cost-effectiveness analyses historically. Such calculation could have enhanced the precision and generalisability of the analysis. Future studies should address these limitations to enhance the robustness, generalisability, and applicability of cost-effectiveness analyses across different healthcare settings.

## Conclusions

Our study highlights the cost-effectiveness of TIVA and sub-Tenon block with Dexamethasone in preventing POV in paediatric strabismus surgery, with the addition of Ondansetron not providing a sufficient economic advantage. These findings suggest that although Ondansetron may offer efficacy benefit in reducing POV incidence, its high cost may limit its use from an economic perspective. Tailoring anaesthesia strategies to both clinical and economic factors remains crucial for optimising outcomes in paediatric strabismus surgery.

## Funding statement

This research received no specific grant from any funding agency in the public, commercial, or not-for-profit sectors.

## Authors’ contributions

Study concept and design: SO, TK, YY

Acquisition, analysis, and interpretation of clinical data: all authors

Drafting of the manuscript: SO

Critical revision of the manuscript for important intellectual content: all authors

Cost-effectiveness analysis: SO

Obtained funding: SO, RT

Supervision: SO, TK

Full access to all the data in the study and responsibility for the integrity of the data and the accuracy of the data analysis: SO, TK

## Declarations of interest

The authors declare that they have no conflicts of interest.

## Figures and Tables

**figs1:**