# Supplementary material for: Cost-effectiveness analysis of anaesthesia regimens for paediatric strabismus surgery based on multicentre retrospective cohort data from Japan
Source: BJA Open. 2025 May 7;14:100404. doi: 10.1016/j.bjao.2025.100404 (PMC12138403; doi:10.1016/j.bjao.2025.100404)
Supplement: Multimedia component 1 [file mmc1.docx]

**Supplementary Table 1. Anesthesia regimens implemented at the three institutions and incidence of post-operative vomiting per anesthesia regimen**

| Anesthetics for maintenance | Intraoperative use of opioids (iv) | Prophylactic use of anti-emetic medications | Application of sub-Tenon block by ophthalmologists | Number of patients | Number of patients suffering POV within 24 hours | Incidence of POV | | The eligible anesthesia regimens for cost effective analysis in this study | |
| --- | --- | --- | --- | --- | --- | --- | --- | --- | --- |
|  |  |  | yes / no | N | N |  | (95% CI) | |  |
| Category 1:  VMA with N_2_O | pentazocine | none | no | 157 | 49 | 0.312 | (0.261, 0.363) | | **Regimen 1** |
|  | pentazocine | none | yes | 11 | 2 | 0.182 | (0.031, 0.333) | |  |
|  | fentanyl | none | no | 12 | 5 | 0.417 | (0.153, 0.681) | |  |
|  | fentanyl | DEX | no | 13 | 1 | 0.077 | (0.000, 0.254) | |  |
|  | fentanyl | DEX | yes | 34 | 4 | 0.118 | (0.018, 0.218) | |  |
|  | no opioids | none | no | 16 | 2 | 0.125 | (0.017, 0.233) | |  |
| Category 2: VMA without N_2_O | pentazocine | none | no | 146 | 34 | 0.233 | (0.181, 0.285) | | **Regimen 2** |
|  | pentazocine | none | yes | 12 | 3 | 0.25 | (0.027, 0.473) | |  |
|  | fentanyl | none | no | 59 | 7 | 0.119 | (0.057, 0.181) | | **Regimen 3** |
|  | fentanyl | DEX | no | 33 | 6 | 0.182 | (0.048, 0.316) | |  |
|  | fentanyl & remifentanil | none | no | 24 | 6 | 0.25 | (0.082, 0.418) | |  |
|  | fentanyl & remifentanil | DEX | yes | 12 | 1 | 0.083 | (0.000, 0.246) | |  |
| Category 3: TIVA | fentanyl | DEX | yes | 61 | 4 | 0.066 | (0.016, 0.116) | | **Regimen 4** |
|  | fentanyl | DEX, OND | yes | 20 | 0 | 0 | (0.000, 0.000) | |  |
|  | remifentanil | DEX | yes | 12 | 0 | 0 | (0.000, 0.000) | |  |
|  | fentanyl & remifentanil | none | yes | 75 | 14 | 0.187 | (0.126, 0.248) | | **Regimen 5** |
|  | fentanyl & remifentanil | DEX | yes | 1,229 | 111 | 0.09 | (0.079, 0.101) | | **Regimen 6** |
|  | fentanyl & remifentanil | DEX, OND | yes | 326 | 12 | 0.037 | (0.016, 0.058) | | **Regimen 7** |
| Category 4: CIVIA with N_2_O | pentazocine | none | no | 44 | 11 | 0.25 | (0.131, 0.369) | | **Regimen 8** |
|  | pentazocine | DEX | no | 11 | 1 | 0.091 | (0.000, 0.255) | |  |
|  | fentanyl | DEX | no | 16 | 0 | 0 | (0.000, 0.000) | |  |
|  | fentanyl | DEX | yes | 88 | 6 | 0.068 | (0.014, 0.122) | | **Regimen 9** |
| Category 5: CIVIA without N_2_O | pentazocine | none | no | 34 | 9 | 0.265 | (0.161, 0.369) | |  |
|  | fentanyl | DEX | no | 22 | 1 | 0.045 | (0.000, 0.133) | |  |
|  | fentanyl | DEX | yes | 18 | 2 | 0.111 | (0.015, 0.207) | |  |
|  | fentanyl & remifentanil | DEX | yes | 32 | 1 | 0.031 | (0.000, 0.092) | |  |
|  | no opioids | none | no | 37 | 4 | 0.108 | (0.031, 0.185) | | **Regimen 10** |

**Note:** Regimens with fewer than 10 cases were excluded from the analysis due to the uncertainty in POV incidence. Although some regimens used ondansetron alone as an intraoperative prophylactic antiemetic, these regimens had fewer than 10 cases each. Therefore, regimens using ondansetron alone were excluded from the analysis.

**Abbreviations:** CI: confidence interval, CIVIA: combined inhalational and intravenous anaesthesia, DEX: dexamethasone, IV: intravenous, N_2_O: nitrous

oxide, OND: ondansetron, POV: post-operative vomiting, TIVA: total intravenous anaesthesia, VMA: volatile maintenance anaesthesia.
