# Supplementary material for: Cost-effectiveness analysis of anaesthesia regimens for paediatric strabismus surgery based on multicentre retrospective cohort data from Japan
Source: BJA Open. 2025 May 7;14:100404. doi: 10.1016/j.bjao.2025.100404 (PMC12138403; doi:10.1016/j.bjao.2025.100404)
Supplement: Multimedia component 2 [file mmc2.docx]

**Supplementary Table 2. Descriptive statistics for each anaesthesia regimen**

|  | Number of Patients | Age | Body weight | Anaesthesia time | Surgery time | Propofol for maintenance | Pentazocine | Fentanyl | Remifentanil | DEX | OND |
| --- | --- | --- | --- | --- | --- | --- | --- | --- | --- | --- | --- |
| Unit |  | [years] | [kg] | [min.] | [min.] | [mg kg^-1^ min.^-1^] | [mg] | [mcg kg^-1^ hour^-1^] | [mcg kg^-1^ min.^-1^] | [mg] | [mg] |
| AR 1 | 157 | 6 (4, 9) | 21.0  (16.0, 31.5) | 57.0  (48.0, 69.0) | 26.0 (21.0, 37.5) | - | - | - | - | - | - |
| AR 2 | 146 | 6 (4, 9) | 20.1  (16.0 29.9) | 54.0  (46.0, 64.3) | 25.0 (15.8, 35.0) | - | 5.50  (1.50, 6.25) | - | - | - | - |
| AR 3 | 59 | 7 (6, 12) | 22.2  (19.5, 45.4) | 84.0  (70.0, 97.0) | 43.0 (35.0, 58.0) | - | - | 2.42  (1.96, 2.89) | - | - | - |
| AR 4 | 61 | 6 (5, 8) | 20.4  (17.6, 30.1) | 57.0  (47.5, 72.5) | 30.0 (17.0, 42.5) | 7.03  (5.61, 8.78) | - | 2.04  (1.52, 2.42) | - | 3.30  (3.30 3.30 | - |
| AR 5 | 75 | 6 (4, 9) | 20.5  (16.6, 29.0) | 66.0  (55.0, 80.0) | 30.0 (19.0, 40.0) | 5.50  (4.71, 6.28) | - | 2.09  (1.62, 2.68) | 0.121  (0.106, 0.137) | - | - |
| AR 6 | 1,229 | 8 (6, 11) | 25.2  (19.4, 37.7) | 68.0  (57.0, 81.0) | 31.0 (23.0, 44.0) | 5.53  (4.74, 6.31) | - | 2.02  (1.57, 2.60) | 0.13  (0.104, 0.139) | 3.30  (3.30, 4.95) | - |
| AR 7 | 326 | 7 (5, 11) | 24.2  (19.3, 36.5) | 72.5  (61.0, 87.0) | 38.0 (28.0, 50.0) | 5.72  (4.91, 6.57) | - | 2.24  (1.65, 2.84) | 0.134  (0.116, 0.152) | 3.30  (3.30, 3.30) | 2.40  (2.00, 3.70) |
| AR 8 | 44 | 6 (5, 7) | 18.0  (16.2, 23.2) | 58.0  (46.3, 69.0) | 25.0 (17.3, 41.0) | 1.20  (0.91, 1.37) | 4.50  (3.00, 5.63) | - | - | - | - |
| AR 9 | 88 | 6 (4, 11) | 21.9  (18.0, 36.7) | 61.0  (51.3, 74.0) | 26.0 (19.3, 35.8) | 4.51  (3.29, 5.31) | - | 1.77  (1.35, 2.35) | - | 3.30  (3.30, 6.60) | - |
| AR 10 | 37 | 6 (5, 9) | 20.1  (17.0, 27.5) | 53.0  (41.5, 66.5) | 29.0 (22.0, 47.0) | 2.94  (2.16, 3.48) | - | - | - | - | - |

**Note:** Values are presented as Median (Interquartile Range [IQR], 25th-75th percentile). For these parameters, the ranges used in the one-way sensitivity analyses correspond to the IQR (25th-75th percentile), and the distributions employed in the probabilistic sensitivity analyses were based on a gamma distribution.

**Abbreviation:** AR: anaesthesia regimen, DEX: dexamethasone, OND: ondansetron
