# Supplementary material for: Cost-effectiveness analysis of anaesthesia regimens for paediatric strabismus surgery based on multicentre retrospective cohort data from Japan
Source: BJA Open. 2025 May 7;14:100404. doi: 10.1016/j.bjao.2025.100404 (PMC12138403; doi:10.1016/j.bjao.2025.100404)
Supplement: Multimedia component 3 [file mmc3.docx]

**Legends to Supplementary Figures**

**Supplementary Figure 1**

**Title: Flow diagram of eligible cases for data analyses**

**Legend:** Between February 2016 and November 2023, 3,571 children underwent strabismus surgery. After excluding 799 patients (e.g. under 3 years old and over 16 years old, second and subsequent strabismus surgery, gene abnormality, ASA-PS III/IV/V, desflurane usage), 2,772 children were included in the analysis. Among the eligible cases in the analysis, the distribution was as follows: Institution 1 - 67.6%, Institution 2 - 25.9%, Institution 3 - 6.5%. A total of 2,222 patients (80.2% of eligible patients) received one of the top 10 most common anaesthesia regimens, numbered Regimen 1 to Regimen 10. Only three TIVA regimens with use of propofol, fentanyl and remifentanil were included in the primary analysis based on the fact that ondansetron was only used in this specific anaesthesia regimen in our clinical data for this study.

**Abbreviations:** ASA-PS: American Society of Anaesthesiologists Physical Status Classification, TIVA: total intravenous anaesthesia

**Supplementary Figure 2**

**Title: Decision tree model for cost-effectiveness analysis**

**Legend:** A decision tree (DT) model was constructed using TreeAge Pro Healthcare 2024 software (TreeAge Pro Inc., Williamston, Massachusetts, the U.S.) to compare the various anesthesia regimes for pediatric strabismus surgery. The DT was employed to model the various outcomes associated with different anaesthetic regimens. This method involved creating a tree structure where each branch represented a possible outcome based on the choice of anaesthetic and subsequent interventions. The DT model was preferred over a Markov model due to the binary nature of the decisions involved in this study (i.e., the occurrence of POV or not), which did not require the time-dependent state transitions typically associated with Markov models. The analysis was conducted from a Japanese public healthcare payer perspective, which includes direct healthcare costs but excludes indirect costs such as POV-related family productivity losses. Direct costs considered were anaesthetic drugs, antiemetic medications, and treatments for POV. Surgery, anaesthesia, and medical staff fees were not separately calculated, as they were assumed consistent across all regimens. For rescue treatment in cases of POV, the cost of antiemetic medications (e.g., metoclopramide) and intravenous infusion treatments were included in the calculation. As per the standard of care in the three participating hospitals, all patients undergoing strabismus surgery were assumed to be hospitalised overnight, regardless of whether or not they experienced POV. Intravenous cannulas were maintained in situ after surgery for all patients. The main health outcome of interest was the prevention of POV. Based on data from the three institutions and existing literature, a DT was constructed to set the "averted POV (aPOV)" as the outcome, calculated as: aPOV = 1 − POV rate.

**Abbreviations:** POV: postoperative vomiting, POV_P: POV positive, POV_N: POV negative, pPOV: probability of POV, cSevo: cost of sevoflurane, cN2O: cost of nitrous oxide, cPent: cost of pentazocine, cMCP: cost of metoclopramide, cFluids; cost of intravenous fluids, cFent:cost of fentanyl, cRopiv: cost of ropivacaine, cDex: cost of dexamethasone, c: Ond: cost of ondansetron, uAverted_POV: utility of averted POV

**Supplementary Figure 3**

**Title: Tornado diagram for one-way sensitivity analysis comparing the ICER of Regimen 6 (TIVA+DEX) versus Regimen 7 (TIVA+DEX+OND)**

**Legend:** The ICER was most sensitive to the POV rate in Regimen 7 (TIVA+DEX+OND), followed by the cost of OND and the POV rate in Regimen 6 (TIVA+DEX).

**Abbreviations:** DEX: dexamethasone, EV: expected value, ICER: incremental cost-effectiveness ratio, OND: ondansetron, POV post-operative vomiting, TIVA: total intravenous anaesthesia, WTP: willingness to pay

**Supplementary Figure 4**

**Title: Incremental cost-effectiveness plane for probabilistic sensitivity analysis comparing the ICER of Regimen 6 (TIVA+DEX) versus Regimen 7 (TIVA+DEX+OND)**

**Legend:** The ICER scatterplot shows that most points fall within the third quadrant, where the incremental cost is negative and incremental effectiveness is also negative. This suggests that Regimen 7 is less cost-effective compared to Regimen 6, as it leads to higher costs without significant improvements in effectiveness. Only a small proportion of simulations (0.1%) fall within the superior quadrant, indicating rare instances where Regimen 7 is more effective and cost-efficient.

**Abbreviations:** WTP: willingness-to-pay, ICER: incremental cost-effectiveness ratio, POV post-operative vomiting, TIVA: total intravenous anaesthesia, DEX: dexamethasone, OND: ondansetron

**Supplementary Figure 5**

**Title: Cost-effectiveness acceptability curves for probabilistic sensitivity analysis comparing all the top 10 regimens**

**Legend:** A recommended anesthesia regimens varies from regimen if the threshold of WTP varies.

**Abbreviations:** WTP: willingness to pay
