# Supplementary material for: Cost-effectiveness analysis of anaesthesia regimens for paediatric strabismus surgery based on multicentre retrospective cohort data from Japan
Source: BJA Open. 2025 May 7;14:100404. doi: 10.1016/j.bjao.2025.100404 (PMC12138403; doi:10.1016/j.bjao.2025.100404)
Supplement: Multimedia component 4 [file mmc4.pptx]

## Slide 1
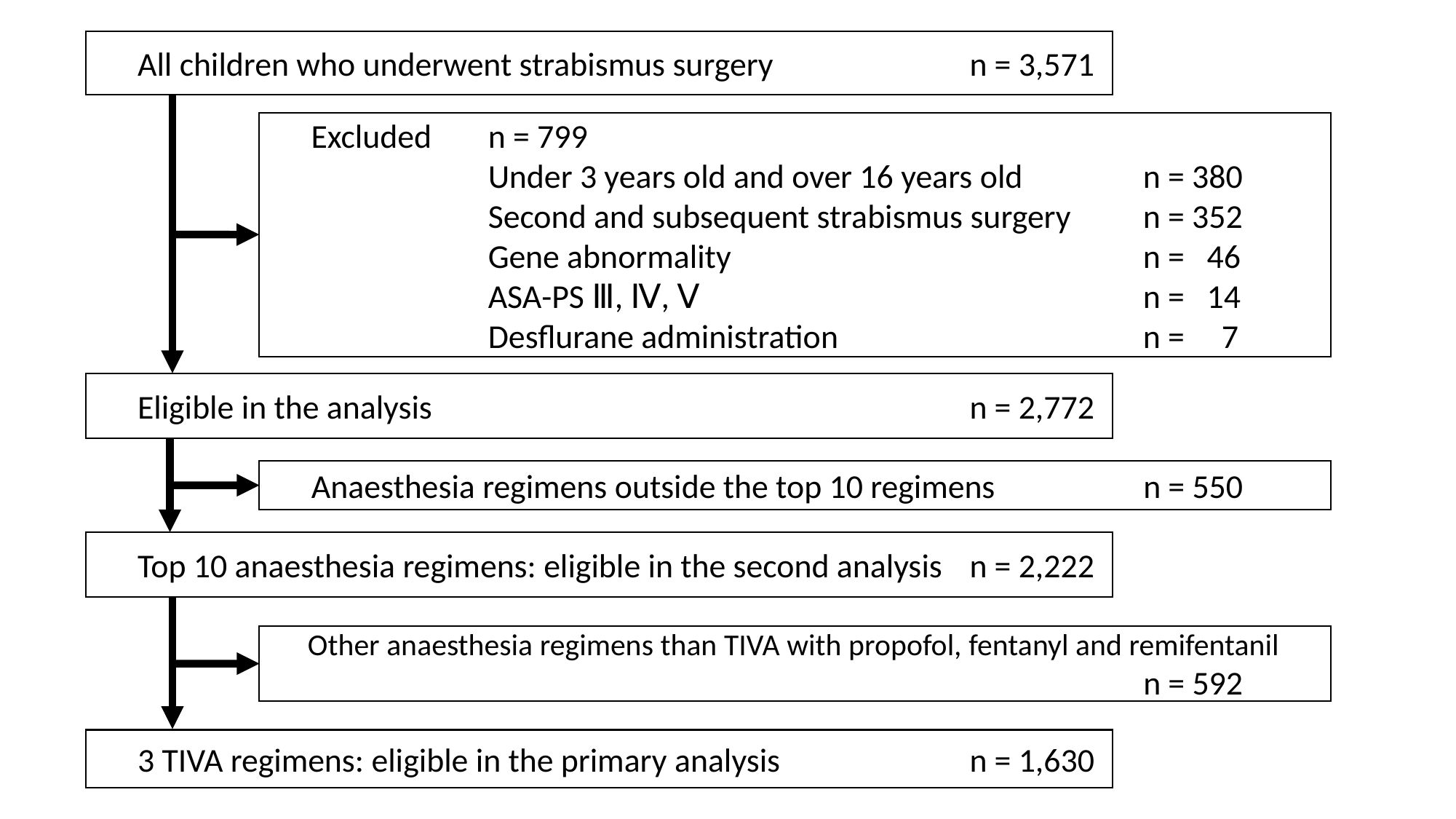

All children who underwent strabismus surgery		n = 3,571
　Excluded	n = 799
 		Under 3 years old and over 16 years old		n = 380　		Second and subsequent strabismus surgery	n = 352
 		Gene abnormality				n = 46
		ASA-PS Ⅲ, Ⅳ, Ⅴ 				n = 14
		Desflurane administration			n = 7
　Eligible in the analysis					n = 2,772
　Anaesthesia regimens outside the top 10 regimens		n = 550
　Top 10 anaesthesia regimens: eligible in the second analysis	n = 2,222
　Other anaesthesia regimens than TIVA with propofol, fentanyl and remifentanil								n = 592
　3 TIVA regimens: eligible in the primary analysis		n = 1,630
