# Supplementary material for: Cost-effectiveness analysis of anaesthesia regimens for paediatric strabismus surgery based on multicentre retrospective cohort data from Japan
Source: BJA Open. 2025 May 7;14:100404. doi: 10.1016/j.bjao.2025.100404 (PMC12138403; doi:10.1016/j.bjao.2025.100404)
Supplement: Multimedia component 5 [file mmc5.pptx]

## Slide 1
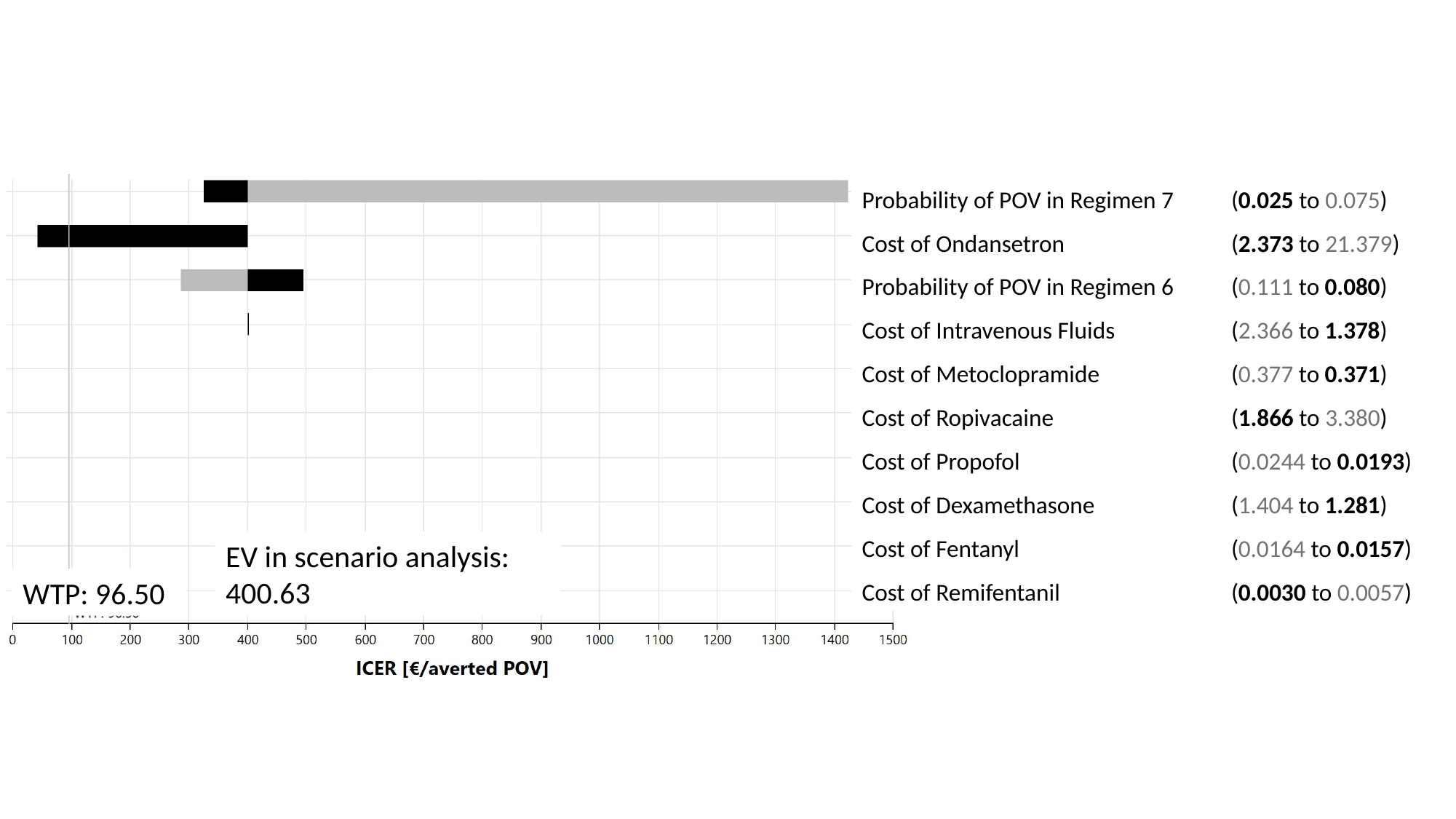

(0.025 to 0.075)
(2.373 to 21.379)
(0.111 to 0.080)
(2.366 to 1.378)
(0.377 to 0.371)
(1.866 to 3.380)
(0.0244 to 0.0193)
(1.404 to 1.281)
(0.0164 to 0.0157)
(0.0030 to 0.0057)
Probability of POV in Regimen 7
Cost of Ondansetron
Probability of POV in Regimen 6
Cost of Intravenous Fluids
Cost of Metoclopramide
Cost of Ropivacaine
Cost of Propofol
Cost of Dexamethasone
Cost of Fentanyl
Cost of Remifentanil
EV in scenario analysis:
400.63
WTP: 96.50
