# Supplementary figures and images for: Cost-effectiveness analysis of anaesthesia regimens for paediatric strabismus surgery based on multicentre retrospective cohort data from Japan
Source: BJA Open. 2025 May 7;14:100404. doi: 10.1016/j.bjao.2025.100404 (PMC12138403; doi:10.1016/j.bjao.2025.100404)

## Slide 1
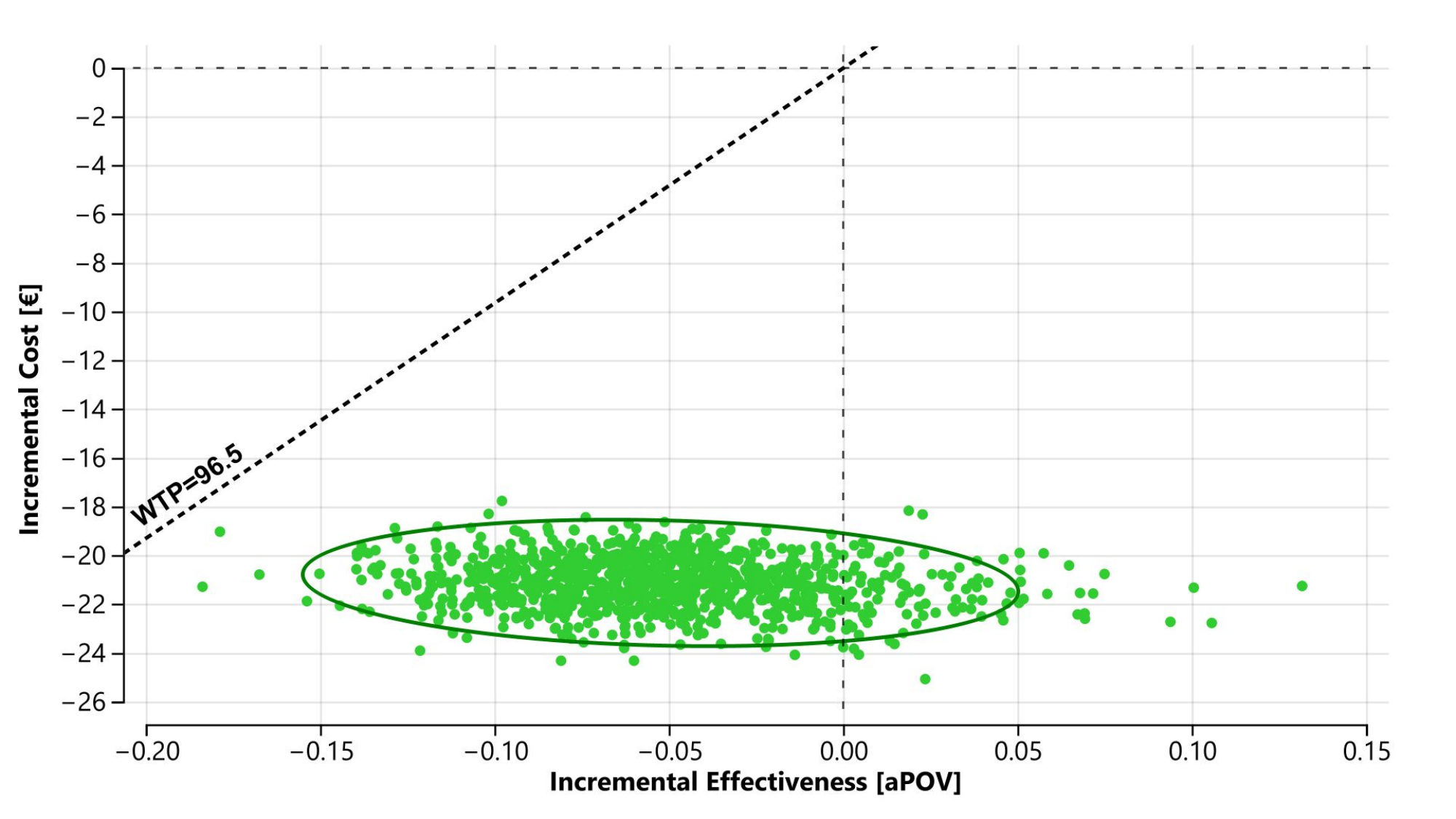

Supplement: Multimedia component 6 [file mmc6.pptx]

## Slide 1
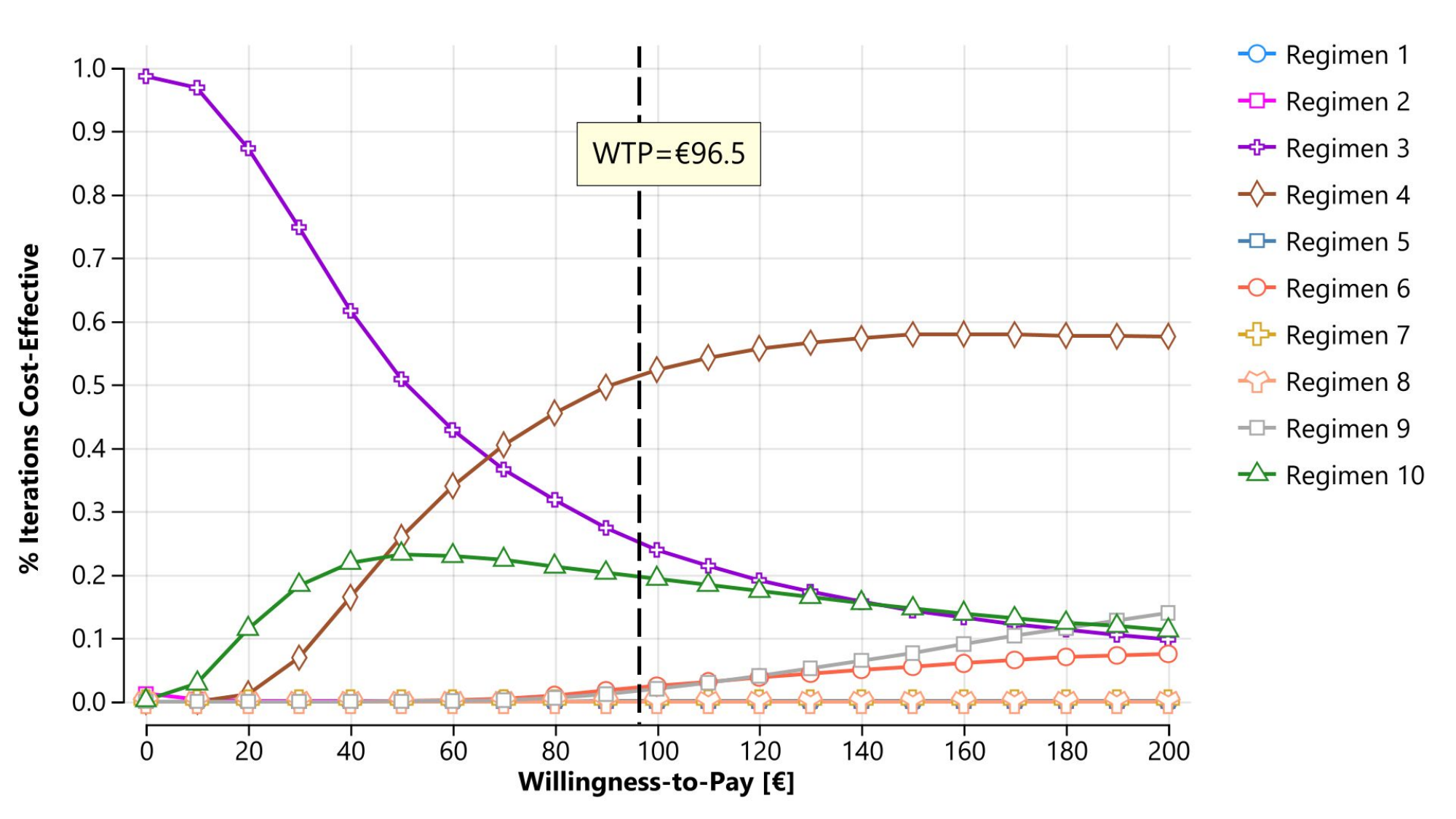

Supplement: Multimedia component 7 [file mmc7.pptx]
